# Olmsted Syndrome

**DOI:** 10.1155/2012/927305

**Published:** 2012-12-23

**Authors:** Renata Elise Tonoli, Damiê De Villa, Renata Hübner Frainer, Luana Pizzarro Meneghello, Nelson Ricachnevsky, Maurício de Quadros

**Affiliations:** ^1^Department of Dermatology, Brazilian Society of Dermatology, Santo Antônio Hospital, Santa Casa de Midericórdia de Porto Alegre, Porto Alegre, RS, Brazil; ^2^Pediatric Dermatology Service, Department of Dermatology, Brazilian Society of Dermatology, Santo Antônio Hospital, Santa Casa de Midericórdia de Porto Alegre, Porto Alegre, RS, Brazil

## Abstract

Olmsted syndrome is a rare congenital, sharply circumscribed transgredient palmoplantar keratoderma. It was first described by Olmsted in 1927. The diagnosis of this rare disease depends on clinical features like symmetrical involvement of keratoderma of the palms and soles and the symmetrical hyperkeratotic plaques around the body orifices. It starts in the neonatal period or in childhood. The disease has a slow but progressive and extremely disabling course. Treatment of Olmsted syndrome is often based on topical therapy with retinoic acid, corticosteroid, emollients, and keratolytics. The present paper describes a case of Olmsted syndrome and its treatment.

## 1. Case Report

**Figure 1:**
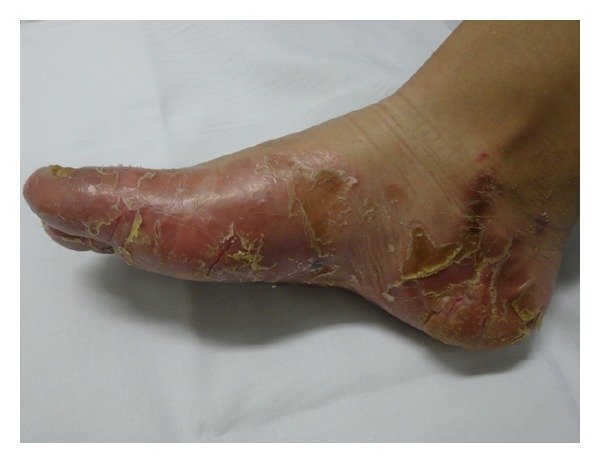
Plantar keratoderma.

**Figure 2:**
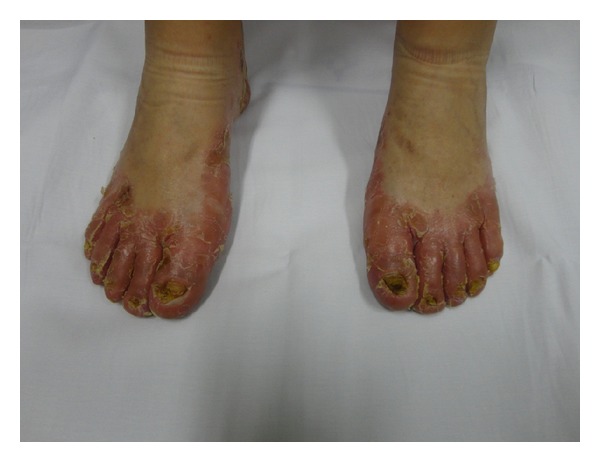
Erythema and fissured fingers.

**Figure 3:**
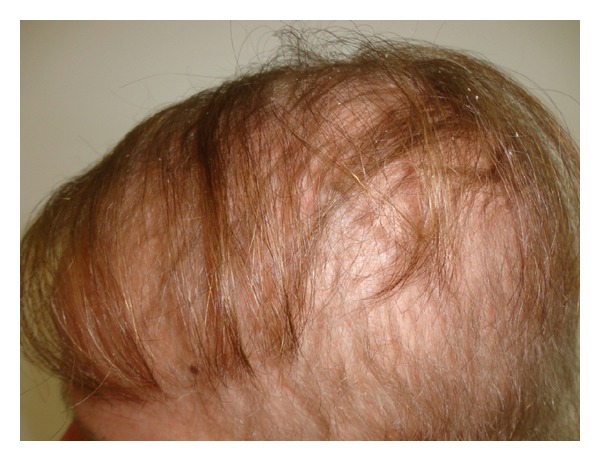
Diffuse alopecia.

**Figure 4:**
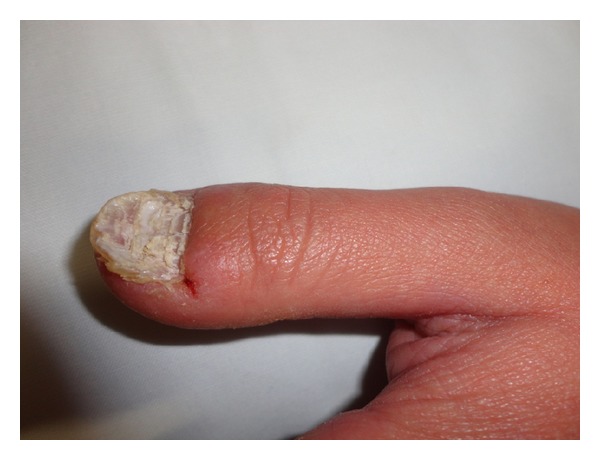
Nail dystrophy.

**Figure 5:**
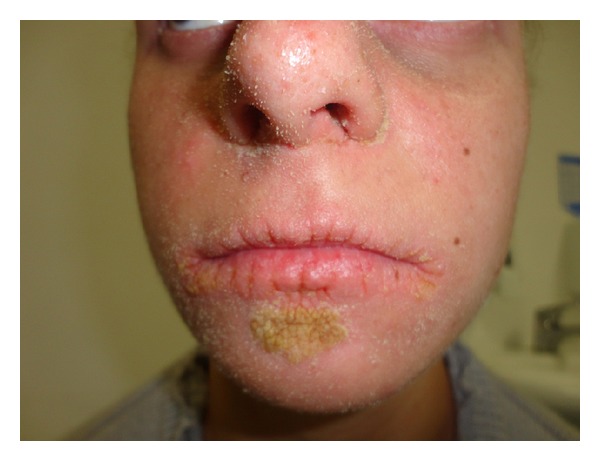
Hyperkeratotic plaque on chin, perioral fissures.

**Figure 6:**
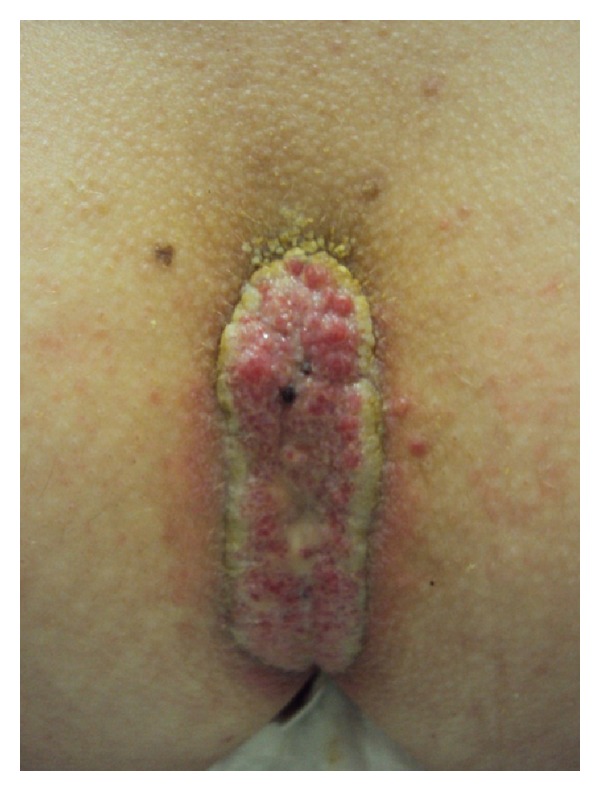
Perianal plaque.

**Figure 7:**
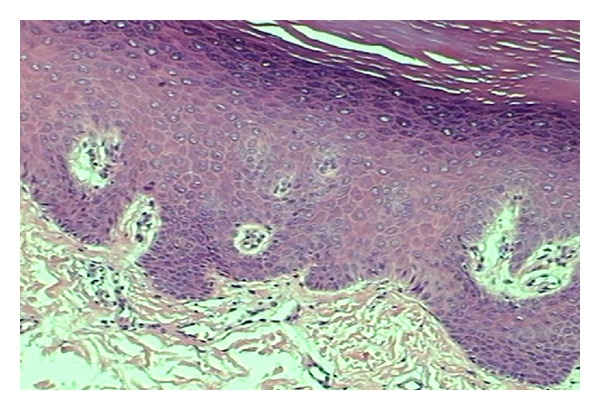
Histopathological exam.

**Figure 8:**
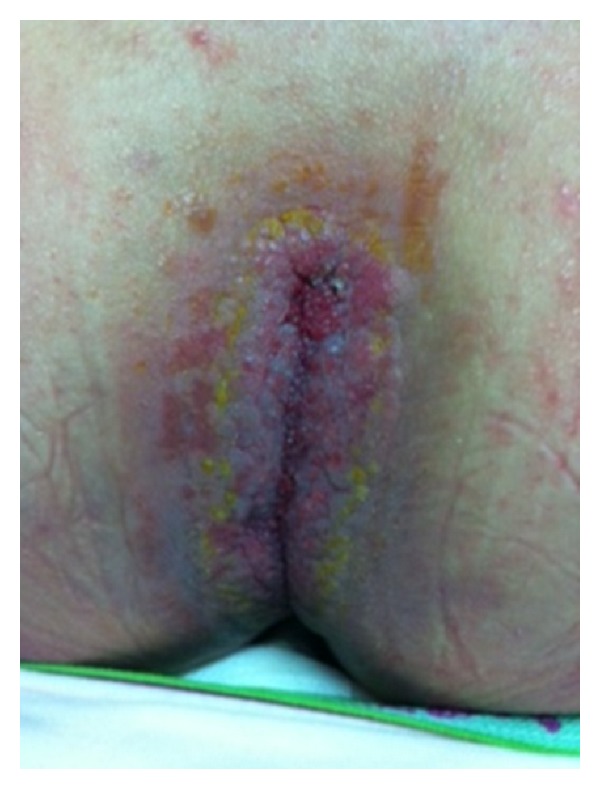
Improvement of perianal hyperkeratotic plaque after treatment.

A 16 year-old female child presented with palmoplantar keratoderma, fissured lips, and mild scaling of the buttocks, fissures in the fingers and foot, perianal, vaginal, and nasal mucosa erosions, diffuse alopecia and sparse hair, nail dystrophy, and perianal hyperkeratotic plaque (Figures 1, 2, 3, 4, 5, and 6). The patient also has a congenital deafness, delayed speech, and development and learning difficulties. The skin biopsy demonstrated psoriasiform epidermal hyperplasia with marked hyperkeratosis, and mild acantopapilomatosis, focal porokeratosis, hypergranulosis and superficial perivascular inflammatory infiltration (Figure 7). The immunostaining showed ki67 (mib-1) positive in 45% of the basal cells and CKM (AE1AE3) strongly positive in suprabasal layer. According to the clinical presentation, biopsy and immunohistochemical studies results of the diagnosis of Olmsted syndrome were made. The treatment was made with keratolytics and emollients initially without improvement. We decided how her life's quality was not good starting with systemic retinoid (acitretin) with a moderate clinical and life's quality improvement after one year with this treatment (Figure 8). 

## 2. Discussion

Olmsted syndrome is a rare congenital disorder, characterized by bilateral sharply circumscribed transgredient palmoplantar keratoderma and periorificial keratotic plaques. Palmoplantar keratoderma may lead to flexural deformities and spontaneous amputation of the fingers [1–5]. It was first described by Olmsted in 1927. Until recently, only 46 individuals had been reported, including 36 sporadic cases and four families containing ten affected individuals. The definitive mode of inheritance was still uncertain, and autosomal-dominant, X-linked-dominant, and X-linked-recessive modes of inheritance had been proposed [3–5].

There are studies (Lin et al.) that demonstrate the gain of function mutations within TRPV3 on chromosomal region 17p13, which encodes a transient receptor potential vanilloid-3 cation channel and give rise to the Olmsted syndrome phenotype. This gain of function mutations might lead to elevated apoptosis of keratinocytes and consequent skin hyperkeratosis in the affected individuals. Studies have suggested that TRPV3 plays a role in skin keratinization, hair growth, and possibility itching sensation in humans and selectively targeting TRPV3 could provide therapeutic potential for keratinization disorders [5].

Cytokeratins have been identified as abnormal in the skin affected by keratoderma that consisted of staining involving the entire thickness of the epidermis with cytokeratin AE1 (normally this cytokeratin only stains the basal layer of the epidermis). Normally cytokeratin 10 is confined to the suprabasal layers whereas this cytokeratin 10 stained only the upper layer of the epidermis in hyperkeratotic lesions of Olmsted syndrome. Some studies showed that expressions of cytokeratins 5 and 14 were abnormally increased similar to those of hyperproliferative disorders. These cytokeratin abnormalities may indicate that the skin involved in Olmsted syndrome retains an immature proliferative profile. Immunohistochemical studies with Ki-67 marker demonstrated that hyperproliferative activity involves the basal and suprabasal keratinocytes in Olmsted syndrome [1, 4]. 

The diagnosis of the disease depends on clinical features [4, 5]. The two major ones are the symmetrical involvement of keratoderma of the palms and soles and the symmetrical hyperkeratotic plaques around the body orifices. It starts in the neonatal period or in childhood [2–5]. The disease has a slow but progressive and extremely disabling course [1, 3, 4].

Other clinical manifestations of Olmsted syndrome include leukokeratosis of the tongue or the oral mucosa, diffuse alopecia, sparse hair, nail dystrophy, hyperhidrosis of the palms and soles, hypohidrosis, hyperkeratotic linear streaks on the elbows, knees, axillae, and the antecubital fossae. Multiple systemic associations have been reported; these include growth retardation, dental anomalies, hearing loss, and corneal opacities.

There are reports of squamous cell carcinoma and malignant melanoma developed in the area of palmoplantar keratoderma [1, 3, 4]. In addition, perianal and inguinal plaques frequently show maceration and infection by bacteria and *Candida albicans *[4]. The differential diagnosis includes other syndromes of palmoplantar keratoderma and hyperkeratotic syndromes [2–5].

Treatment of Olmsted syndrome is often disappointing. Topical therapy with retinoic acid, corticosteroid, emollients, and keratolytics may offer temporary symptomatic relief. Systemic retinoids may be effective but are often only partially so or not helpful at all. Our patient had a moderate improvement with this treatment. We believe that systemic retinoids might be tried when others therapies do not work out and lesions interfere in quality of life.
